# A scoping review of COVID-19 experiences of people living with dementia

**DOI:** 10.17269/s41997-021-00500-z

**Published:** 2021-04-06

**Authors:** Juanita-Dawne R. Bacsu, Megan E. O’Connell, Claire Webster, Lisa Poole, Mary Beth Wighton, Saskia Sivananthan

**Affiliations:** 1 Department of Psychology, Rural Dementia Action Research (RaDAR) Team, Canadian Centre for Health and Safety in Agriculture (CCHSA), University of Saskatchewan, Saskatoon, Saskatchewan S7N 5A5 Canada; 2 Department of Psychology, Canadian Centre for Health and Safety in Agriculture, University of Saskatchewan, Arts 182, 9 Campus Drive, Saskatoon, SK S7N 5A5 USA; 3 Caregiver Crosswalk Inc., Montreal, Canada; 4 Dementia Advocacy Canada, Toronto, Canada; 5 Alzheimer Society of Canada, Toronto, Canada

**Keywords:** Coronavirus, COVID-19, Dementia, Alzheimer’s disease, Synthesis, Coronavirus, COVID-19, démence, maladie d’Alzheimer, synthèse

## Abstract

**Objectives:**

Compared with the general population, people living with dementia have been unequivocally affected by the COVID-19 pandemic. However, there is a paucity of knowledge on the COVID-19 impact on people with dementia and their care partners. The objective of this scoping review was to synthesize the existing literature on the COVID-19 experiences of people with dementia and their care partners.

**Methods:**

Following Arksey and O’Malley’s scoping review framework, we searched five electronic databases (Scopus, PubMed, CINAHL, EMBASE, and Web of Science) and an online search engine (Google Scholar). Inclusion criteria consisted of English-language articles focusing on the COVID-19 experiences of people with dementia and their care partners.

**Synthesis:**

Twenty-one articles met our inclusion criteria: six letters to the editor, seven commentaries, and eight original research studies. In the literature, five main themes were identified: (i) care partner fatigue and burnout; (ii) lack of access to services and supports; (iii) worsening neuropsychiatric symptoms and cognitive function; (iv) coping with COVID-19; and (v) the need for more evidence-informed research. Factors such as living alone, having advanced dementia, and the length of confinement were found to exacerbate the impact of COVID-19.

**Conclusion:**

Urgent action is needed to support people living with dementia and their care partners in the pandemic. With little access to supports and services, people with dementia and their care partners are currently at a point of crisis. Collaboration and more evidence-informed research are critical to reducing mortality and supporting people with dementia during the pandemic.

## Résumé

**Objectifs:**

Comparativement à la population générale, les personnes vivant avec la démence ont incontestablement été touchées par la pandémie de COVID-19. On en sait toutefois peu sur l’effet de la COVID-19 sur les personnes atteintes de démence et leurs partenaires soignants. Notre étude de champ visait à résumer la littérature existante sur l’expérience de la COVID-19 chez les personnes atteintes de démence et leurs partenaires soignants.

**Méthode:**

En suivant le cadre méthodologique d’Arksey et O’Malley pour les études de champ, nous avons interrogé cinq bases de données électroniques (Scopus, PubMed, CINAHL, EMBASE et Web of Science) et un moteur de recherche en ligne (Google Scholar). Nous avons inclus tous les articles en anglais portant sur l’expérience de la COVID-19 chez les personnes atteintes de démence et leurs partenaires soignants.

**Synthèse:**

Vingt et un articles ont correspondu à nos critères d’inclusion : six lettres publiées dans le courrier des lecteurs, sept commentaires et huit études de recherche originales. Cinq grands thèmes sont ressortis de ces articles : i) la fatigue et l’épuisement professionnel des partenaires soignants; ii) le manque d’accès aux services et aux mesures d’aide; iii) l’aggravation des symptômes neuropsychiatriques et des fonctions cognitives; iv) les façons de faire face à la COVID-19; et v) le besoin de plus de recherche fondée sur les preuves. Nous avons constaté que trois facteurs, soit le fait de vivre seul(e), la démence avancée et la durée du confinement, exacerbaient l’effet de la COVID-19.

**Conclusion:**

Une action urgente est nécessaire pour aider les personnes vivant avec la démence et leurs partenaires soignants durant la pandémie. N’ayant guère accès aux mesures d’aide et aux services, les personnes atteintes de démence et leurs partenaires soignants se trouvent actuellement dans une situation de crise. La collaboration et la recherche fondée sur les preuves sont essentielles pour réduire la mortalité et aider les personnes atteintes de démence durant la pandémie.

## Introduction

The current coronavirus disease (COVID-19 from the SARS-CoV-2 virus) is causing global morbidity and mortality, with a disproportionate burden on people living with dementia. Recent data show that dementia is the most common comorbidity in COVID-19-related deaths (O’Brien et al. 2020). In Canada, 85% of all COVID-19 deaths have been in long-term care facilities, where two thirds of people have dementia (Alzheimer’s Disease International 2020b; Suárez-González et al. 2020). In the United Kingdom, the Office for National Statistics (ONS) reported that 50% of deaths in care homes from COVID-19 have been people with dementia (Alzheimer’s Society 2020).

In response to COVID-19, several countries have imposed social distancing restrictions and lockdown measures in attempts to reduce the spread of the virus (Alzheimer’s Disease International 2020a). These measures have included restrictions on social gatherings, limited mobility outside of the home, and restrictions and/or closure of all non-essential services such as home care services and health clinic access (Brown et al. 2020). However, these ‘protective’ measures are having adverse outcomes on people with dementia and their care partners (Anderson and Parmar 2020; McGhan and McCaughey 2020).

Without access to social supports and healthcare services, people living with dementia are experiencing greater risks not only from COVID-19 itself, but also from social isolation. More specifically, social isolation is known to increase risk of premature death from all causes (National Academies of Sciences, Engineering, and Medicine 2020). In England, the number of people dying at home from dementia and Alzheimer’s disease has increased by 79.3% during the pandemic compared with the previous 5-year average (Office for National Statistics 2020). While some excess deaths may be related to missed COVID-19 diagnoses (e.g., misattribution of COVID-19 deaths to pneumonia or other respiratory illnesses, or issues of delayed reporting of COVID-19 deaths), there may also be deaths from other factors associated with the pandemic (Fineberg 2020; Woolf et al. 2020). Researchers suggest that COVID-19 confinement and social isolation may be contributing to the rapid deterioration of people with dementia and increased morbidity (Migliaccio and Bouzigues 2020; Killen et al. 2020). Despite this knowledge, there is a paucity of information on the COVID-19 experiences of people with dementia and their family care partners. However, understanding the impact of COVID-19 (e.g., challenges and coping strategies) is essential to informing policies and programs to support people with dementia and their care partners during the pandemic. Accordingly, the purpose of this review was to synthesize the existing literature on the experiences of COVID-19 on people with dementia and their family care partners.

## Research design and methods

This study was guided by Arksey and O’Malley’s (2005) scoping review framework that consists of five stages: (i) identifying the research question; (ii) searching for relevant studies; (iii) selecting the studies; (iv) charting the data; and (v) summarizing and reporting the results. While systematic reviews assess the quality of the research, a scoping review maps the literature on an emerging topic and identifies knowledge gaps to inform policy and research (Arksey and O’Malley 2005).

### Identifying the research question

The objective of this scoping review was to synthesize the existing literature on the experiences of COVID-19 on people affected by dementia. More specifically, the review question was: What are the experiences of people living with dementia and their family care partners during the COVID-19 pandemic?

### Search strategy

Relevant articles were retrieved by searching five electronic databases including Scopus, PubMed, CINAHL, EMBASE, and Web of Science. Google Scholar was also searched to identify other relevant articles. The keywords used for the search are shown in Table 1. The search timeline focused on articles published between January 13 and September 15, 2020. January was selected as the start date for the timeline as public health officials confirmed the first reported case of COVID-19 outside of China on January 13, 2020 (World Health Organization 2020).

**Table 1:** Keyword search strategy

Concept	Keywords	Databases and search engines
Coronavirus	COVID-19* OR Coronavirus Infection* OR Coronavirus Infection Disease* OR 2019-nCoV Infection* OR SARS-CoV-2* OR Coronavirus Disease 2019* OR COVID*	Scopus, PubMed, CINAHL, EMBASE, Web of Science, and Google Scholar
Dementia	Dementia* OR Alzheimer’s disease* OR Alzheimer’s* OR Lewy Bodies* OR Lewy Body Dementia* OR Frontotemporal* OR Vascular* OR Parkinson’s Disease*	

### Inclusion and exclusion criteria

Articles were included in this review if they met five inclusion criteria: (i) original research, commentaries/editorials, or letters to the editor; (ii) written in the English language; (iii) full-text, peer-reviewed journal articles; (iv) published between January 13 and September 15, 2020; and (v) focused on the COVID-19 experiences of people with dementia (e.g., community-dwelling and/or care facilities) and/or their family care partners. Exclusion criteria consisted of studies that were (i) published in languages other than English; (ii) not published between January 13 and September 15, 2020; and (iii) not focused on the review’s objective but rather clinical outcomes in terms of the incidence and mortality of dementia comorbidities in COVID-19 patients.

### Study selection and data charting

**Fig. 1:**
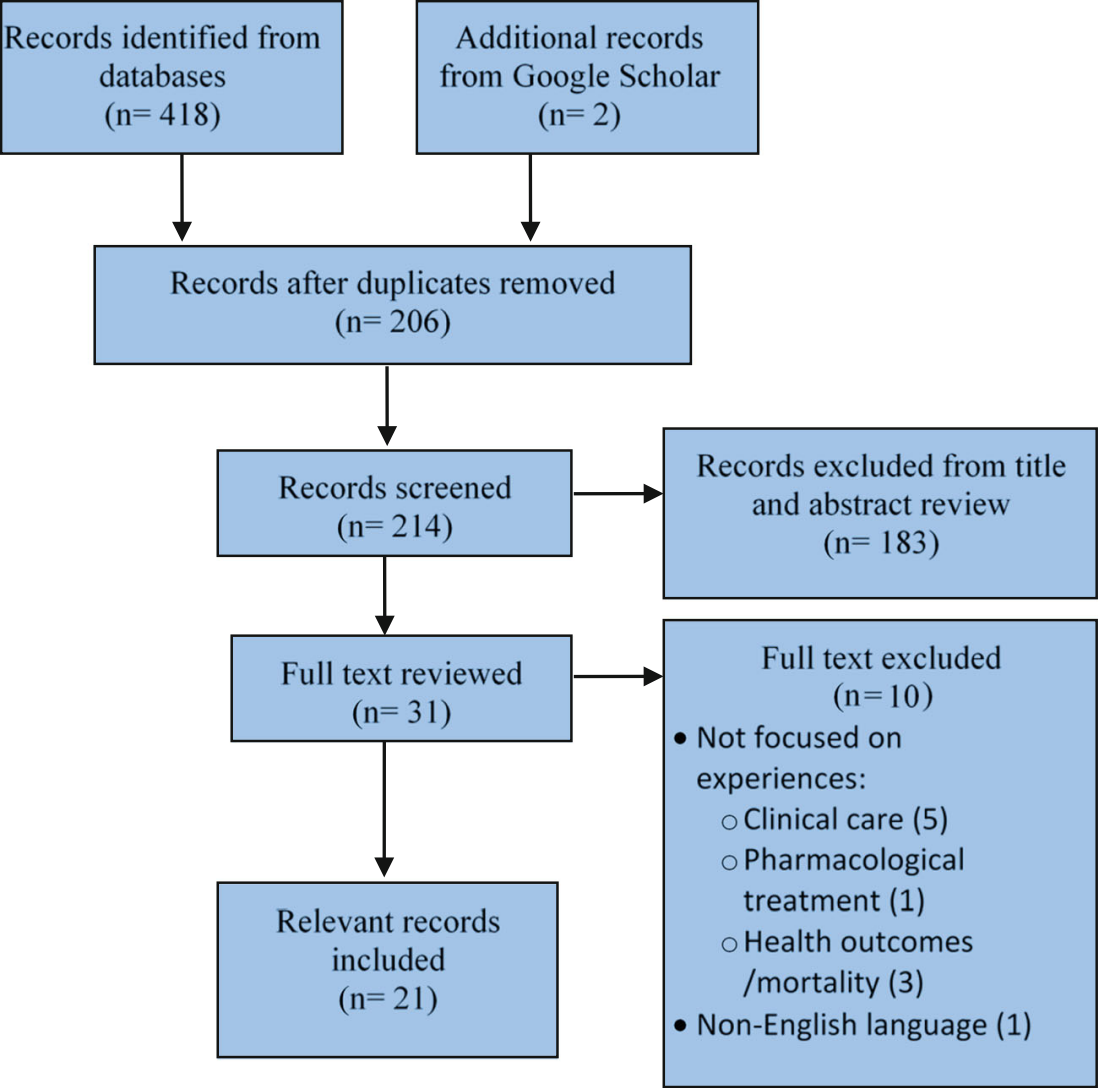
Study flow chart

This search identified 420 articles for possible inclusion in the review. Relevant search findings were imported into the bibliographic management software RefWorks, followed by a systematic de-duplication. After 206 duplicates were removed, the titles and abstracts of 214 articles were screened for relevancy independently by two reviewers. A total of 183 articles were excluded as they did not meet the inclusion criteria. Any discrepancies or uncertainty related to the data inclusion were discussed between the two reviewers. The full texts of the remaining 31 articles were reviewed, and 10 articles were excluded based on the full-text assessment. The final number of articles included in the scoping review was 21. Figure 1 shows the flowchart of the literature search and screening process. The articles were charted into a table using the following information: article type; authors; country; purpose; methods; sample; and findings. A description of the 21 articles is provided in Table 2.

**Table 2:** Selected articles

Article type	Authors	Country	Purpose	Methods	Sample/residence	Findings
Original research	Boutoleau-Bretonniere et al.	France	Understand effects of confinement on people with Alzheimer’s disease (AD)	Quantitative questionnaire	38 patients with AD and 38 family care partners Community dwelling	Confinement negatively impacts neuropsychiatric (depression, anxiety and agitation) symptoms and cognition in AD patients with low baseline cognitive function.
Original research	Cohen et al.	Argentina	Impact of confinement oncare partner stress andwell-being	Quantitative questionnaire survey	80 family care partners Community dwelling	Confinement increased care partner stress, severity depended on PwD’s levels of cognition. PwD (people living with dementia) discontinued cognitive/physical therapies, and had more anxiety.
Original research	Goodman-Casanova et al.	Spain	Impact of confinement onthe health and well-beingof people with mildcognitive impairment (MCI)or mild dementia	Quantitative telephone survey	93 participants with mild dementia or MCI Community dwelling	People living alone reported more negative psychological effects (anxiety, less well-being, negative feelings) and sleeping problems. Supports included support networks for food/medications, daily routines, social interaction, staying informed, and physical activity.
Original research	Lai et al.	China	Telemedicine to mitigateimpact of confinementon PwD and caregivers	Quantitative interviews andquestionnaires	60 older adults with dementia and care partner-dyads Community dwelling	Care partners with telehealth intervention had varying improvements in physical and mental health, perceived burden, and self-efficacy compared with group without intervention. PwD who did not receive the telehealth intervention had lower neurocognitive functioning and quality of life.
Original research	Lara et al.	Spain	Impact of lockdown on neuropsychiatric symptoms and quality of life	Quantitative questionnaire	40 patients with a diagnosis of mild cognitive impairment or AD Community dwelling	After 5 weeks of lockdown, study found worsening neuropsychiatric symptoms in patients with AD and MCI, with agitation, apathy and aberrant motor activity being the most affected symptoms.
Original research	Roach et al.	Canada	Impact and lived-experiences of social and physical distancing during COVID-19	Qualitative interviews	21 participants including PwD and family care partners Community dwelling	PwD experienced decreased mental health (anxiety, fear, worry) and more cognitive decline from COVID-19 confinement. Care partners reported issues of mental health and burnout. Challenges included loss of informal and formal supports, lack of health care, social isolation, and care partner concerns of returning to work without supports for PwD.
Original research	Savla et al.	USA	Family care partners’ appraisal of stressors in confinement	Mixed methods telephone survey	53 rural family care partners of PwD Community dwelling	Care partner challenges included restrictions on daily routines, limited socialization, and reduced or terminated services (respite care). Care partner coping supports included gardening, alone time, going outside, making masks, spending time on cell phones and playing computer games.
Original research	Vaitheswaran	India	Experiences and challenges of care partners of PwD during lockdown	Qualitative interviews	31 care partners of PwD Community dwelling	Most PwD had increasing behavioural and psychological symptoms (anxiety, upset about masks, sleeping issues, and being disruptive to others at home). Care partner challenges included closure of formal supports, challenges with technology, managing chores/supplies, not having access to dementia specialists, financial challenges, difficulty getting dementia medications, fear of PwD getting COVID-19 and becoming institutionalized.
Letter to Editor	Barros et al.	Portugal	Addressing the needs of PwD and care partners during COVID-19	N/A	N/A	Challenges for PwD and care partners included: cancelled activities, day care centres, and care-related services; visiting prohibited in nursing homes; and no formal supports during lockdown.
Letter to Editor	Canevelli et al.	Italy	Identifying challenges and supports to mitigate impact of COVID-19 on PwD and care partners	N/A	N/A	Challenges for PwD and care partners included lack of social support, lack of formal care, and care partner burnout. Supports included technology for cognitive/social stimulation, daily routines, doing at-home activities (gardening, reading and exercise),and food/pharmacy delivery services.
Letter to Editor	Padala et al.	USA	Facetime to reduce behavioural problems during COVID-19 confinement	Qualitative case study	81-year-old nursing home resident with AD Nursing home	No contact order led to more depression, anxiety, apathy, irritability, difficulty sleeping, and restlessness. After Facetime, staff reported PwD was less anxious and agitated and had improved appetite.
Letter to Editor	Palmero et al.	Italy	Explore impact of confinement on cognition in people with Parkinson’s disease (PD)	Quantitative questionnaire	28 people with PD with varying levels of cognitive impairment and dementia Community dwelling	Challenges included loss of support systems, increased physical inactivity, and disrupted daily routines. Majority reported worsening of anxiety and cognitive symptoms (memory and attention).
Letter to Editor	Velayudhan et al.	UK	Mental health experiences of PwD in care homes during COVID-19	N/A	N/A	Enforced isolation among PwD in care homes may contribute to anxiety, agitation, depression, boredom, physical inactivity, and a decreased quality of life. Need for innovative interventions and research on non-pharmacological approaches for neuropsychiatric symptoms in PwD in care homes.
Letter to Editor	Wang et al.	China	Impact of COVID-19 on PwD	N/A	N/A	Challenges for PwD included loss of services, difficulty adhering to social distancing and good hygiene, and barriers to accessing telemedicine.
Commentary	Brown et al.	Canada	Anticipating/mitigating the impact of COVID-19 on PwD	N/A	N/A	Challenges for PwD included social isolation, confinement, lack of physical exercise, suspension of purposeful activity and reduced social engagement. Care partners may become ill, may need to isolate, or may develop anxiety and mental health issues. Technological innovations needed to support dementia research and develop non-pharmacological interventions that can be delivered at home (cognitive training, social interaction, physical exercise).
Commentary	Chen	Ireland	Supporting quality of life for PwD during COVID-19	N/A	N/A	PwD may have more mental health and neuropsychiatric symptoms after COVID-19 lockdown. Innovation needed to support PwD such as creative at-home activities, technology (social interaction/access to health services), teleconsultations for medication refills.
Commentary	Cheung et al.	New Zealand	Challenges and technology to support PwD during COVID-19	N/A	N/A	Study found positive effects from using a virtual cognitive stimulation therapy for PwD during COVID lockdown.
Commentary	Edelman	USA	Mitigating COVID-19 impacts and improving nursing home care for PwD	N/A	N/A	Challenges for PwD in nursing homes included social isolation, limited physical activity, behavioural issues, and changes in routine. Need for more and improved technologies for social support and improved nursing home care.
Commentary	Greenberg et al.	USA	Impact of COVID-19 on PwD and care partners	N/A	N/A	Challenges for PwD included disrupted routines, decreased respite care, and safety issues (violence and hygiene). Increased burden on care partners without home care (tube feeding, injections, home dialysis, and catheter care). Increased need for technology to foster social support (Facetime, WhatsApp) and research on COVID-19 effects on PwD and care partners.
Commentary	Killen	UK	COVID-19 challenges on PwD and care partners	N/A	N/A	Challenges included decreased physical health, worsening cognitive and neuropsychiatric symptoms, mental health issues, and care partner fatigue.
Commentary	Migliaccio	France	COVID-19 confinement on PwD and their care partners	N/A	N/A	Challenges of COVID-19 on PwD and care partners range from physical health to psychological health.

*AD*, Alzheimer's disease; *PwD*, people living with dementia; *MCI*, mild cognitive impairment; *PD*, Parkinson’s disease

## Results

From the 21 articles identified in the review, 6 were letters to the editor, 7 were commentaries, and 8 were original research. The articles were from Argentina (*n* = 1), Canada (*n* = 2), China (*n* = 2), France (*n* = 2), Ireland (*n* = 1), Italy (*n* = 2), New Zealand (*n* = 1), Spain (*n* = 2), Portugal (*n* = 1), India (*n* = 1), the UK (*n* = 2), and the United States (*n* = 4).

In the literature on COVID-19 experiences of people with dementia and their care partners, five main themes were identified: (i) care partner fatigue and burnout; (ii) lack of access to services and supports; (iii) worsening neuropsychiatric symptoms and cognitive function; (iv) coping with COVID-19; and (v) the need for more evidence-informed research.

(i) Care partner fatigue and burnout

Studies indicate that COVID-19 restrictions and imposed measures of confinement have added significant strain to the pre-existing workload of family care partners of people with dementia (Cohen et al. 2020; Vaitheswaran et al. 2020). More specifically, ten articles discussed issues of care partner fatigue and burnout (Barros et al. 2020; Brown et al. 2020; Canevelli et al. 2020; Cohen et al. 2020; Greenberg et al. 2020; Killen et al. 2020; Migliaccio and Bouzigues 2020; Roach et al. 2020; Savla et al. 2020; Vaitheswaran et al. 2020). For example, COVID-19 restrictions have increased care partners’ workloads by limiting and/or terminating access to home care, respite, daycare programs, meal programs, medical specialists, health services, and supports (Cohen et al. 2020; Roach et al. 2020).

The COVID-19 pandemic has intensified the pre-existing issue of limited supports and services available for care partners of people with dementia (Savla et al. 2020). In the literature, specific challenges experienced by family care partners during COVID-19 included increased household chores, limited social interaction (Savla et al. 2020), restricted movement outside the home, employment disruptions, financial concerns (Roach et al. 2020), technological challenges (Vaitheswaran et al. 2020), medication management for persons with dementia (Brown et al. 2020), and restricted/terminated formal supports during the lockdown (e.g., respite care and home care services) (Barros et al. 2020). Consequently, care partners experienced increased frustration and work overload because they were solely responsible for providing care (Cohen et al. 2020; Savla et al. 2020).

Greenberg and colleagues (Greenberg et al. 2020) assert that without home care, care partners face increased challenges by having to perform complex medical tasks such as tube feeding, colostomy and catheter care, injections, and home dialysis. Accordingly, issues related to the care partner’s mental health (Brown et al. 2020; Roach et al. 2020), physical health (Migliaccio and Bouzigues 2020), and burnout were predominant throughout the literature (Barros et al. 2020; Cohen et al. 2020; Killen et al. 2020l; Canevelli et al. 2020; Savla et al. 2020; Vaitheswaran et al. 2020). Moreover, studies suggest a correlation between the level of care partner workload and the length of at-home/community-dwelling confinement, especially for care partners of people with advanced dementia (Cohen et al. 2020; Vaitheswaran et al. 2020).

(ii) Lack of access to services and supports

Several articles described the challenges of the COVID-19 lockdown and closure of non-essential businesses on people living with dementia. For example, Roach et al. (2020) assert that the COVID-19 lockdown severely impacted people living with dementia by altering their daily routines, limiting physical activity, increasing social isolation, and restricting and/or terminating health care services and supports. Similarly, other authors suggest that COVID-19 confinement contributed to increased physical inactivity, a lack of purposeful activities, limited intellectual stimulation, and increased social isolation for people living with dementia (Brown et al. 2020).

In discussing the challenges of COVID-19 confinement, many authors described limited access to health services and programs. More specifically, these services included reduced or terminated access to day programs (Palermo et al. 2020), respite care (Savla et al. 2020), cognitive stimulation programs (Lara et al. 2020), meal programs, home care services (Greenberg et al. 2020), neurological rehabilitation therapy (Cohen et al. 2020), physical activity programs (Brown et al. 2020; Edelman et al. 2020; Roach et al. 2020), health clinics (Killen et al. 2020; Migliaccio and Bouzigues 2020), and dementia care specialists (Vaitheswaran et al. 2020).

The confinement from COVID-19 and physical distancing measures also contributed to social support challenges for people with dementia. Boutoleau-Bretonniere et al. (2020) assert that social gatherings, volunteer events, and even religious activities were cancelled, which impacted persons living with dementia. Moreover, people with dementia living in long-term care homes were recognized as being especially vulnerable to the isolating effects of COVID-19 (Edelman et al. 2020). Several articles noted that enforced isolation in long-term care homes has contributed to an increase in loneliness, depression, boredom, apathy, anxiety, irritability, restlessness, and difficulty sleeping (Padala et al. 2020), and to an overall decreased quality of life for people with dementia (Velayudhan et al. 2020).

(iii) Worsening neuropsychiatric symptoms and cognitive function

Twelve of the twenty-one articles addressed the impact of COVID-19 confinement on people with dementia’s neuropsychiatric symptoms and cognitive function (Barros et al. 2020; Boutoleau-Bretonniere et al. 2020; Canevelli et al. 2020; Chen and Chen 2020; Cohen et al. 2020; Goodman-Casanova et al. 2020; Lai et al. 2020; Lara et al. 2020; Palermo et al. 2020; Roach et al. 2020; Vaitheswaran et al. 2020; Wang et al. 2020). For example, Boutoleau-Bretonniere et al. (2020) found that confinement negatively affected neuropsychiatric symptoms (e.g., depression, anxiety, and agitation) and cognition in people with Alzheimer’s disease with low pre-pandemic cognitive function. Similarly, Vaitheswaran and colleagues (2020) reported increasing behavioural and psychological symptoms in people with dementia, such as disruptive behaviours, symptoms of anxiety, irritability, expressions of being upset about wearing facemasks or others wearing facemasks, and sleep disturbances. Other articles also suggested worsening neuropsychiatric symptoms in confinement, such as agitation, apathy (Lara et al. 2020), anxiety (Chen and Chen 2020; Cohen et al. 2020), depression (Velayudhan et al. 2020), sleep disorders (Canevelli et al. 2020), and deteriorating cognitive function (Barros et al. 2020; Lai et al. 2020; Palermo et al. 2020; Roach et al. 2020). In particular, people with dementia who lived alone (Goodman-Casanova et al. 2020; Wang et al. 2020) or who had lower levels of cognition were identified as being more vulnerable to experiencing negative neuropsychiatric effects from the lockdown (Boutoleau-Bretonniere et al. 2020; Cohen et al. 2020).

(iv) Coping with COVID-19

Despite the numerous articles documenting the COVID-19 challenges, only four articles discussed the coping strategies used by people with dementia and their care partners. A study on rural family care partners identified different coping strategies, including gardening, having alone time, being outdoors, making protective masks for care aides, spending time on their cell phones, and playing computer games (Savla et al. 2020). Three other articles described coping strategies used to address confinement by persons with dementia and their care partners, including maintaining daily routines with chores and leisure activities (e.g., reading, computer games, and exercise), having family support networks and/or delivery services to access groceries and medications, having social interaction through telephone or video chats (Brown et al. 2020; Canevelli et al. 2020), and staying informed by following the news (Goodman-Casanova et al. 2020). Key to many of these descriptors of coping was engagement with technology.

(v) Need for evidence-informed research

In discussing COVID-19, eleven of the articles identified the need for more evidence-informed research on home-based interventions to support people with dementia during the pandemic (Brown et al. 2020; Chen and Chen 2020; Cheung and Peri 2020; Edelman et al. 2020; Greenberg et al. 2020; Lai et al. 2020; Canevelli et al. 2020; Migliaccio and Bouzigues 2020; Padala et al. 2020; Vaitheswaran et al. 2020). For example, the literature highlighted the need for more research on the implementation (e.g., accessibility, ease of use, affordability, and barriers) of home-based technological interventions such as physical exercise programs (Brown et al. 2020), cognitive stimulation therapy (Cheung and Peri 2020), and the expansion of telemedicine services (e.g., home video consultations, prescription refills, and virtual access to health specialists) during the pandemic (Edelman et al. 2020; Lai et al. 2020; Canevelli et al. 2020; Chen and Chen 2020; Vaitheswaran et al. 2020).

While technological interventions are not new, home-based interventions are relatively newly available to people with dementia and their care partners (Padala et al. 2020). Consequently, people with dementia who live alone (Brown et al. 2020) or care partners may struggle with technology and require instruction, online training, and tools to support their usage of technology (Barros et al. 2020; Vaitheswaran et al. 2020). As such, Cheung and Peri (2020) suggest that future research is necessary to understand the implementation and technological challenges from the perspective of people with dementia and their care partners.

Articles also highlighted the need for more research on the implementation and evaluation of virtual social support interventions for people with dementia and their care partners (Migliaccio and Bouzigues 2020). For example, Greenberg et al. (2020) note that social interaction can be facilitated by smartphone technology and computer applications such as FaceTime and WhatsApp. Given the lockdown and visiting restrictions within institutions, technology and social networking platforms were recognized as being especially important for people in long-term care and nursing home facilities (Edelman et al. 2020; Padala et al. 2020). However, Padala et al. (2020) assert that the use of such applications needs to be studied and evaluated to assess the ease of access and usage in institutional care.

## Discussion

People with dementia and their care partners have experienced unprecedented burden and mortality from the COVID-19 pandemic. Increased age, frailty, and health conditions often associated with dementia increase the risk of complications from COVID-19 (Alzheimer Society of Canada 2020; Centers for Disease Control and Prevention 2020). Despite these risks, there are no known attempts to examine the current state of the literature on the impact of COVID-19 (e.g., challenges, barriers, supports and coping strategies) among people living with dementia. However, understanding the COVID-19 impact is essential to informing policies and programs to support people with dementia during the pandemic. Accordingly, the purpose of this scoping review was to identify and synthesize the existing literature on COVID-19 experiences of people with dementia and their family care partners.

This scoping review found that COVID-19 has severely impacted the lives of people with dementia and their care partners (Roach et al. 2020; Savla et al. 2020). For example, our study’s findings suggest that people with dementia are experiencing substantial challenges from COVID-19, such as care partner fatigue and burnout, confinement challenges, and worsening neuropsychiatric symptoms and deteriorating cognitive function. These challenges resonated across the various types of manuscripts examined in this review (e.g., commentaries, letters to the editor, and original research studies). Moreover, factors such as living alone (Goodman-Casanova et al. 2020), having advanced dementia, and the length of community-dwelling confinement were found to exacerbate the detrimental impacts of the pandemic (Boutoleau-Bretonniere et al. 2020; Cohen et al. 2020).

With restricted and/or terminated access to formal services (e.g., home care, meal programs, and respite), family care partners for people with advanced dementia were identified as being particularly vulnerable to experiencing care partner burnout from COVID-19 (Savla et al. 2020). Anecdotally, in our clinical practice, we have observed additional challenges for families living with dementia than those published in the literature. Some examples of these challenges include difficulty recruiting formal/paid help, issues with learning how to manage complex tasks (e.g., incontinence issues, medication management, feeding, and bathing) without educational training, and exacerbated family tension and disagreements regarding care planning for a loved one with dementia.

Findings from this scoping review have important research and policy implications. In terms of directions for research, this review found that there is an urgent need for more research on the COVID-related coping strategies and supports for people living with dementia and their care partners. While many articles identified the challenges of COVID-19, only a handful of articles addressed coping strategies for people living with dementia (Brown et al. 2020; Goodman-Casanova et al. 2020; Canevelli et al. 2020; Savla et al. 2020). With restricted access to services, the pandemic has intensified the pre-existing workload and lack of services for family care partners (Savla et al. 2020). As such, care partners are experiencing increased stress, mental health issues, fatigue, and burnout. Consequently, more evidence-informed research is needed to examine coping strategies and identify best practices to support care partners in the pandemic (Roach et al. 2020; Savla et al. 2020; Vaitheswaran et al. 2020).

In addition, more evidence-informed research is required on home-based interventions (e.g., cognitive therapy, exercise programs, social support activities, and telemedicine) for people with dementia and their care partners. While technological interventions are not new, home-based interventions are relatively newly accessible to care partners. Accordingly, more research is needed to examine the implementation (e.g., accessibility and ease of use), scale-up (e.g., geographical expansion to rural and remote communities), and evaluation of home-based interventions for people with dementia and their care partners.

In developing COVID-19 policies to support persons with dementia, there is a critical need for collaboration and more evidence-informed research involving people with dementia and their care partners. Although several articles (e.g., letters to the editor and commentaries) have been written on COVID-19 and dementia, relatively few research studies have been conducted with people living with dementia. With personal expertise and lived experience of COVID-19, people with dementia can offer valuable knowledge and insight to identify COVID-19 challenges and mitigation strategies in the pandemic. Thus, partnerships and collaborative research are essential to developing effective COVID-19 policies to support people with dementia and their care partners.

## Limitations

This review was limited to English-language, peer-reviewed literature published between January and September 2020. While a rigorous search method was performed, it is possible that relevant literature was excluded in terms of language and timeframe. For example, studies in different languages may provide a more comprehensive understanding of the COVID-19 experiences on people with dementia. Also, this review’s timeframe only captures the literature from the first wave of the COVID-19 pandemic. A follow-up review is required to compare and analyze literature focusing on the second wave of the pandemic.

Another limitation of this review is that we did not search preprint repositories but focused specifically on peer-reviewed journal articles from electronic databases. As such, it is possible that some relevant articles may have been missed. Given the rapidly evolving COVID-19 situation, future reviews may consider searching preprint servers as a means to finding timely research. However, reviews focusing on preprint servers should be conducted with caution as preprints have not undergone peer review, and a recent article suggests a high retraction rate among COVID-19 articles and preprints (Yeo-Teh and Tang 2021).

In addition, there are some limitations related to the generalizability of our study’s findings in terms of gender and residence. In the literature, only two studies briefly addressed gender. For example, women were more often identified as family care partners (Cohen et al. 2020) and experienced higher levels of burden and workload than men (Vaitheswaran et al. 2020). However, more research is needed to examine the COVID-19 impact in relation to sex and gender for people living with dementia and their care partners. More specifically, consideration of COVID-19-related risk and protective factors for men and women may help to inform and accelerate interventions to reduce mortality and improve quality of life for people with dementia. In terms of residence, the majority of the studies focused on community-dwelling people (e.g., individuals living in private residences) with dementia. For example, only one study focused on a person living with dementia in a care facility (Padala et al. 2020). Consequently, future research is needed to examine the COVID-19 impact on people living with dementia in care facilities.

## Conclusion

There is a growing need for more research to address the impact of COVID-19 on people with dementia and their care partners. Compared with the general population, people with dementia are a vulnerable population with risks not only from COVID-19 but also from social isolation and the physical distancing methods used to mitigate the pandemic’s spread. Confinement measures combined with the lack of access to health and support services have severely impacted the lives of people with dementia and their care partners (Savla et al. 2020; Wang et al. 2020). More specifically, the findings from this review suggest that people living with dementia are experiencing substantial challenges from COVID-19, such as care partner fatigue and burnout, confinement challenges, and worsening neuropsychiatric symptoms and deteriorating cognitive function. Despite these COVID-related challenges, only a few articles addressed coping strategies and supports for people with dementia and their care partners. With little access to supports and services, people with dementia and their family care partners are currently at a point of crisis. Urgent action is needed to support people with dementia and their family care partners during the pandemic.

In developing COVID-19 policies to support people with dementia, there is a critical need for collaboration and more evidence-informed research involving people with dementia and their care partners. With personal expertise and lived experience of COVID-19, people with dementia can offer valuable knowledge and insight to address COVID-19 challenges and mitigation strategies. Collaboration and more evidence-informed research are critical to reducing mortality and supporting people with dementia during the pandemic.

## Data Availability

Additional data may be available upon written request to the first author.
